# Provision versus promotion to develop a handwashing station: the effect on desired handwashing behavior

**DOI:** 10.1186/s12889-017-4316-6

**Published:** 2017-05-05

**Authors:** Debashish Biswas, Fosiul Alam Nizame, Tina Sanghvi, Sumitro Roy, Stephen P. Luby, Leanne E. Unicomb

**Affiliations:** 1 Program for Emerging Infections, Infectious Diseases Division, International Centre for Diarrhoeal Disease Research, Bangladesh (icddr,b), 68, Shaheed Tajuddin Ahmed Sarani, Mohakhali, Dhaka 1212, Bangladesh; 2 Enteric and Respiratory Infections Program, Infectious Diseases Division, International Centre for Diarrhoeal Disease Research, Bangladesh (icddr,b), Dhaka, Bangladesh; 3 Alive & Thrive, Dhaka, Bangladesh; 4 Stanford University, Stanford, California, USA

**Keywords:** Handwashing station, Handwashing behavior, Integrated intervention, Complementary feeding, Child nutrition, Bangladesh

## Abstract

**Background:**

Diarrhea prevalence increases from around the time that complementary foods are introduced. Improving caregiver’s hand hygiene during food preparation could reduce complementary food contamination and enteric pathogen transmission. Washing hands with soap is more common when water and soap are together at a convenient location. We conducted a three-month pilot intervention to evaluate two options for setting up handwashing stations: i) provide a handwashing station, or ii) help the family to make their own from available materials. Additionally, we assessed the feasibility of this intervention to be integrated with a child feeding program.

**Methods:**

We conducted the intervention among two groups; 40 households received a free of cost handwashing station and another 40 households were motivated to place their own soap/soapy-water and water vessel near the food preparation and child feeding area. Community health workers encouraged caregivers to wash hands with soap/soapy-water before food preparation and feeding a child. They either assisted study participants to install the study-provided handwashing station at the recommended place or encouraged caregivers to develop their own. Field researchers assessed placement and composition of handwashing stations and the feasibility of integrating handwashing and nutrition messages.

**Results:**

By end of the trial, 39/40 households developed their own handwashing station, comprising a bucket, mug and bar soap/soapy-water of which 60% (6/10) households were observed with a functional and complete handwashing station set. Observed handwashing with soap was detected among 8/10 households from the study-provided handwashing station group and 5/10 among households who had made their own handwashing station. Sixty-seven of the 76 caregivers recalled integrated intervention messages on social and health benefits of infant and young child feeding correctly; and all recalled key handwashing with soap times, before food preparation and feeding a child.

**Conclusion:**

Encouraging households to develop their own handwashing station with soap and water to place at a food preparation/child feeding location is feasible over the short term. In the absence of large-scale provision of handwashing stations, caregivers can be encouraged to create and use their own. Integrating handwashing with soap into a nutrition intervention was feasible and acceptable and should be considered by policy makers.

## Background

Diarrhea and pneumonia continue to be leading causes of death in low income countries [1, 2] and both are more common among children who are malnourished [3–5]. Both growth faltering and diarrhea in low income countries is most marked between 3 and 15 months of age, around the time that complementary foods are introduced [6–9]. When weaning foods are contaminated, enteropathogens can grow exponentially [10, 11]. Hands may be contaminated with fecal matter that can contaminate food [11–13] or they can directly transfer pathogens to adults and children through eating and feeding.

Children in rural Bangladesh whose mothers were observed to wash hands before preparing food, had less diarrhea over the next 2 years compared to children of mother who did not wash hands during food preparation [14]. However, in Bangladesh handwashing with soap before preparing food, before serving food, and before eating is uncommon [15]. WHO recommends that complementary feeding programs should encourage hygienic food preparation and child feeding [16].

Washing hands with soap is more common when water and soap are together at a convenient location [17]. In Bangladesh, water sources and handwashing places are often distant from food preparation areas. In one study in rural Bangladesh, the closest handwashing station at around half (45%) of observed households was more than 10 steps away from the food preparation area, and 65% of household didn’t have a handwashing agent at these locations [18]. Thus, improving convenience and making soap and water available near food preparation and eating areas could enhance handwashing with soap at these times. A handwashing station developed in collaboration with community members in rural Bangladesh, was found acceptable, feasible and suitable in both urban and rural settings [19]. The model aims to provide households with a dedicated location with the water and handwashing agent together, to facilitate handwashing with soap at key times. Understanding behaviors and habit formation when using technology to improve handwashing with soap maximizes the potential of a successful behavior change intervention. This can be described using the Integrated Behavioral Model for Water, Sanitation and Hygiene (IBM-WASH) theoretical framework [20]. The IBM-WASH framework integrates and explains a broad range of factors at multiple levels that affect adoption of behavior related to water, sanitation and hygiene.

We conducted a pilot study to compare the effectiveness of two different strategies that integrated handwashing with soap into a nutrition intervention for the improvement of complementary feeding practices: one group received a study-provided model handwashing station positioned near the area for feeding children and the food preparation area; the other group was encouraged to develop their own handwashing facilities. We also evaluated the feasibility of integrating handwashing with soap and the nutrition intervention.

## Methods

### Study sites and population

**Fig. 1:**
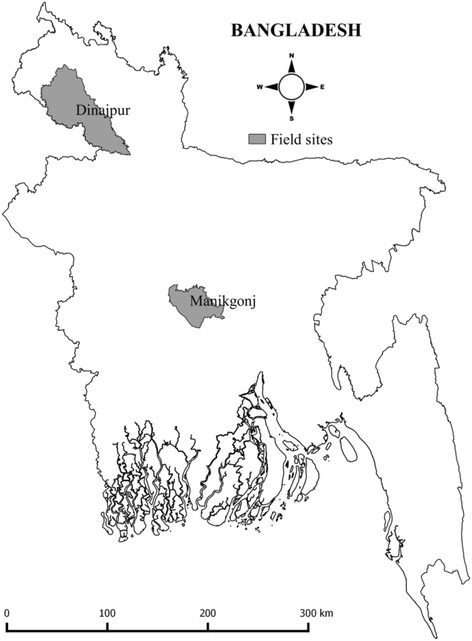
Field sites (drawn by the first author)

This behavior change intervention trial was conducted in two rural districts; Manikgonj (central Bangladesh) and Dinajpur (northern Bangladesh) (Fig. 1). Two sites were used to include different regions of rural Bangladesh. We collected the list of the *upazila*s (sub-districts) from the two districts from the Bangladesh Population Census 2001 [21]. First we excluded urban areas (municipalities and *Pouroshova*) from the *upazila* list and, in order to select our sample proportional to the population, we randomly selected one *upazila* from Manikgonj district and two *upazilas* from Dinajpur. From the three *upazilas* we randomly selected four unions (the smallest rural administrative and local government units in Bangladesh). From each of the four unions we randomly selected one village; thus 4 villages in total were selected from the 3 *upazilas* based on the population size. Within each selected village the field workers identified eligible households based on the criterion of having a child aged between 6 and 23 months who was being fed complementary food. Twenty households from each of the 4 villages were selected through a computer generated random number; 80 households in total.

### Pilot integrated intervention

Alive & Thrive worked to develop a scaled-up intervention for preventing child undernutrition by improving infant & young child feeding (IYCF) practices [22]. Alive & Thrive interventions focused on counseling, coaching, training, and helping mothers use good IYCF practices during home visits following WHO recommendations for complementary feeding and breast feeding [16]. In addition, they held community mobilization forums and meetings in each program village to raise broader awareness about recommended practices and their importance. The program content related to complementary feeding included variety of food, age specific quantity, meal frequency, time of introduction of complementary food, continued breastfeeding, handwashing with soap before food preparation and feeding the child, responsive feeding, feeding during and after illness, and techniques to address poor appetite. Community health workers (CHW) were responsible for conducting home visits and village meetings and forums to discuss these topics. We integrated our promotion of handwashing stations into this IYCF program; the population of the current study was distinct from the final Alive & Thrive IYCF population that comprised their intervention or control sites [22, 23]. The activities were designed on the basis of Alive & Thrive’s existing program on IYCF; and the messages on handwashing were designed on the basis of findings of qualitative and quantitative assessments conducted previously in the same districts [18]. These assessments showed that convenient location of water and soap, belief in the health benefits of handwashing with soap before food preparation and child feeding, and perceptions that social norms (others practice the recommended behaviors) influenced adoption of the recommended handwashing practices. The handwashing station component of the intervention was designed to address the convenience factor. Counseling caregivers during home visits and community mobilization sessions were designed to address belief in benefits and perception of social norms.

Alive & Thrive recruited CHWs to implement the program activities. CHWs belonged to the same community as the primary audience groups and were accepted by the community. They were at least 25 years of age, with those with some experience in delivering health messages were prioritized. Selected CHWs participated in a 3 day training program on IYCF, handwashing and handwashing stations, home visits, and community meetings and forums. A one day practical session involved demonstrating how to construct a handwashing station (with both water and handwashing agent placed together). All eligible households received behavior change promotion about complementary feeding practices and handwashing with soap before food preparation and before feeding the child. The intervention behavior recommendations were: (i) wash hands with soap/soapy-water after defecation, cleaning a child anus, before food preparation and before feeding a child, (ii) maintain and use handwashing station near the food preparation and child feeding area, and (iii) follow the recommendations of age specific quantity, frequency and varieties of food in addition to continued breast feeding.

#### Hardware provision or promotion and instructions

**Fig. 2:**
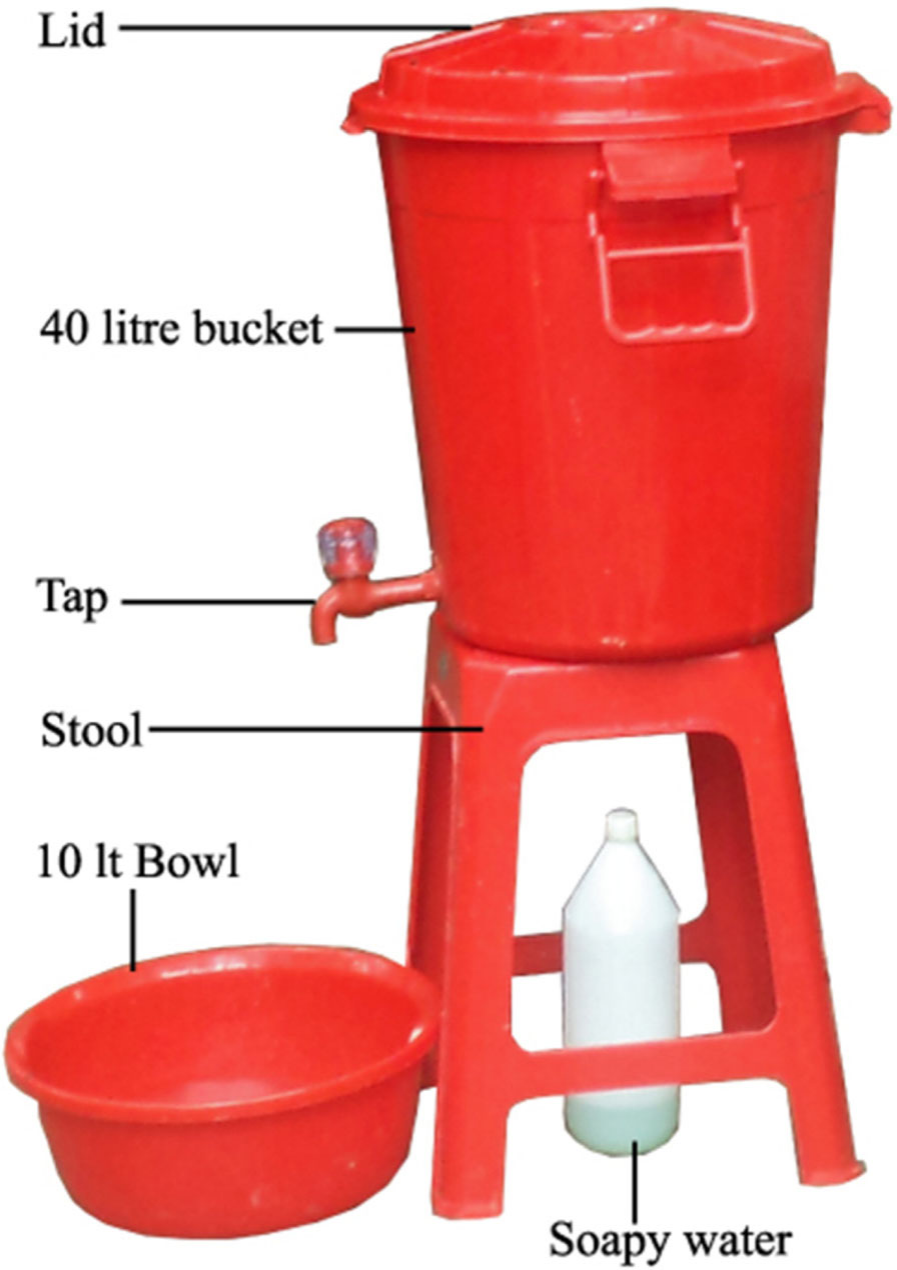
Study provided model handwashing station

For 40 households field staff provided a model handwashing station that comprised a 40 l plastic bucket with tap and lid, a bowl to collect residual rinse water and a stool to use as a stand for the bucket. We also provided a 1.5 l plastic bottle and detergent powder free of charge to make and store soapy-water [19, 24] (Fig. 2). We encouraged the remaining 40 households to prepare their own soapy-water and place their own soap/soapy-water and a vessel to store water for handwashing near the food preparation and child feeding area(s). At each home visit by the CHW, families in both groups were reminded to maintain handwashing stations (both water vessel and handwashing agent are placed appropriately and stocked with water and soap or soapy-water) and wash hands before food preparation and feeding the child. All eligible mothers/caregivers of children aged 6–23 months from the 4 selected villages were the primary audience for the intervention. Fathers, grandfathers, grandmothers and community leaders were prioritized as the secondary audience for influencing behavior change. Trained CHWs conducted 4 follow-up home visits (2 visits per month) during the intervention period for this study.

#### Role of CHWs

**Fig. 3:**
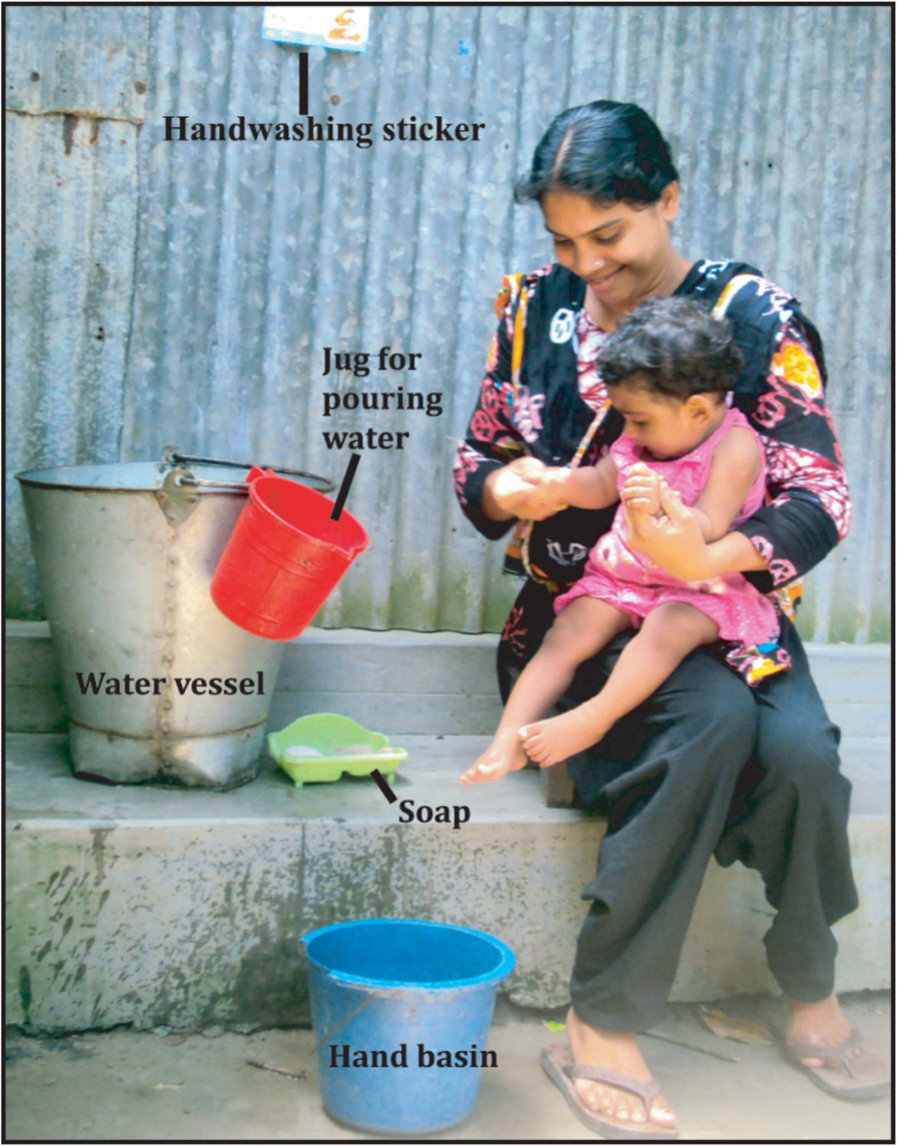
An example of self-made handwashing station

During home visits CHWs assisted the caregivers in installing the study-provided handwashing station for the handwashing station group (study HWS group) and encouraged use. They also demonstrated soapy-water making in these households. CHWs encouraged mothers in the self-made handwashing station group (self-made HWS group) to develop their own handwashing station from available household resources and demonstrated soapy-water making. CHWs asked caregivers to place some type of water vessel but didn’t suggest the need to install a tap as part of their own handwashing station. They encouraged households to place either bar soap or soapy-water near the water vessel (Fig. 3). CHWs worked with study participants to identify a convenient location that would facilitate and remind the caregiver to wash hands before preparing food and before feeding her children, to place the study provided or their own handwashing station. CHWs and their supervisors also conducted mother’s group meetings and community meetings and organized educational videos in the communities.

As part of the integrated IYCF intervention, CHWs distributed handwashing stickers and placed them near the handwashing stations (Fig. 3) as a reminder to wash hands with soap during the recommended key times. Caregivers selected a place to fix the sticker through discussions with the project staff.

TV advertisements that encouraged mothers to wash hands with soap and water before food preparation and child feeding, asked to put water and soap near the place, and messages on complementary feeding were broadcast throughout the intervention period between late September and mid-December, 2011.

### Qualitative data collection

A team of data collectors (field researchers) from icddr,b, separate from the CHWs, conducted qualitative assessments including interviews, observations and group discussions over three rounds at days 20, 58 and 83 after the intervention commenced.

#### Informal interviews

The field research team conducted informal discussions with caregivers at each visit and from each study group. They took detailed notes for each question asked to document their responses. When appropriate, the field researchers included verbatim quotations in the notes that illustrated a particular perspective. The interviewer asked open-ended questions to all eligible caregivers about their perceptions and practices regarding complementary feeding of the child aged 6–24 months, handwashing with soap at the recommended key times, regular use and maintenance of the handwashing station near the food preparation and child feeding area(s), and motivations and barriers to using the handwashing station they received or made themselves.

#### Unstructured observation

Field researchers conducted unstructured observations in 20 randomly selected households (10 households from each HWS group) from within the study population during each of the three follow-up visits. Randomization was done by the lead investigator at icddr,b through computer generated random numbers. Field researchers observed caregiver’s handwashing practices before food preparation and before feeding a child and the use of handwashing stations and soap/soapy-water. Each observation took place from early morning (6 AM) and each session lasted for 2–4 h to cover both recommended key times; e.g. food preparation and child feeding by the family. Field researchers took detailed notes to record actual behaviors.

#### Group discussion

Field researchers conducted 12 group discussions, four at each of the assessment times with equal numbers of family members from each handwashing station group and included one to two elders from the selected households. During the group discussions field researchers discussed family member’s perceptions about handwashing with soap, their experience with handwashing stations and their intention to practice the recommended behaviors.

### Data analysis

Field researchers used an open-ended questionnaire for data collection at each assessment. The data from group discussions were audio recorded and transcribed. Field researchers conducted interviews and discussions in Bengali. On the day of data collection, field researchers translated data to English and entered them into a table with pre-designated headings using Excel, based on the questionnaire. For each question the field researchers included as much information as possible in the table, and we (led by DB and FN) determined the number of persons providing these responses. This was done by including a column for counts (quantifying the qualitative data). On subsequent data collection days, if a response category did not exist for a question, the field researchers added this to the listed responses. We analyzed the interview and group discussion data according to the different behavioral determinants based on the study objectives, at the community level, for handwashing to identify the number of caregivers who were practicing the recommended behavior.

We considered regular handwashing practice (observed) as instances when the household caregiver washed their hands at each time point of the total handwashing opportunities that occurred during the observation period. Similarly, if a complete handwashing station (both water and soap/soapy-water) was found near the recommended places during the observation period and the caregiver used the handwashing station to wash her hand at least once, then we considered this household as a regular user of handwashing station.

We also summarized respondent recall of the IYCF messages for children aged 6–24 months that they heard during the intervention period. Findings from informal interviews, group discussions and observations were compared for consistency and were triangulated. We summarized and compared the responses from the study HWS group and the self-made HWS group. Through comparison and summary, the analysis identified key areas of behavior change and intervention uptake.

## Results

### Socio demographic information

Informal discussions were commonly conducted with mothers, the primary caregivers; 93% (74/80) during the first assessment, 98% (78/80) during the second assessment and 95% (76/80) for the final assessment. The average age of respondents was 24 years in the study HWS group and 23 years in the self-made HWS group. The reported average household income was lower in the study HWS group (BDT. 10,200, US$ 127) than for the self-made HWS group (BDT. 15,700, US$ 196 per month) (Table 1). The educational status for the two groups was similar. The average number of schooling years did not differ between groups (mean = 5.25 and 6.42, *p* = 0.16).

**Table 1:** Demographic information of the study participants

	Study HWS group (*N* = 40)	Self-made HWS group (*N* = 40)
Average age of respondents	23.7	23.2
Household Income per month		
Taka ≤5000 ($63)	16	11
Taka 6000–10,000 ($75–125)	11	10
Taka 11,000–20,000 ($138–250)	11	13
Taka 21,000–30,000 ($263–375)	1	1
Above 30,000 ($375)	2	5
Average of household income (monthly)	US$127	US$196
Mother’s educational level		
No institutional education	9	8
Primary school (I-V)	10	8
High School (VI-X)	19	21
Higher education (XI & above)	2	3
Have own water source	30	37
TV available at home	16	20
Mobile phone available at home	28	36
Radio available at home	3	11
Have electricity at home	21	23

### Self-made handwashing station

By end of the trial, 39/40 households developed their own handwashing station (both water vessel and soap placed together). The self-made handwashing station developed by the community members often comprised an existing household bucket for water storage, a jug or mug to pour water over hands, and a plastic bottle for making and storing soapy-water (Fig. 3). Although a few households placed bar soap with the water storage vessel in addition to soapy-water, all caregivers placed a bottle of soapy-water at the location. Two major factors that motivated caregivers to develop their own handwashing station were ease of use and no cost. For example one caregiver from Dinajpur said, "*I am using a big plastic bucket which was unused for many days. I haven't spent money and that unused casket became a very useful thing. Even this is not a big deal to store water here close to my hands*". Another important factors reported by the caregivers was home visits by community health workers. All mothers received home visits and assistance in setting up a handwashing station with a reminder sticker placed close to the cooking and feeding the child areas. Frequent home visits made caregivers habituated to set up a handwashing station. For example one caregiver from Manikgonj said, "*Our apa* (CHW) *visits our para* (neighborhood) *very frequently and observes whether I placed water or not, even if somehow I met her outside my home she has just one question to me that whether I am using that bucket and washing my hands or not. So it helps me a lot to become habituated day by day".* One barrier for developing a self-made handwashing station was lack of available resources in the households. Some caregivers reported having old vessels that they thought were not suitable for storing water and they used these functional buckets for other purposes. Due to financial difficulties they were unable to buy new vessels to use for handwashing.

### Handwashing practices

Field researchers observed handwashing with soap before food preparation and before feeding a child among 80% (8/10) of caregivers in the study-HWS group and 50% (5/10) from the self-made HWS group at the final assessment. Although there is increasing trends of handwashing with soap in the study-HWS group, greater than for the self-made HWS group, the small sample size did not permit a rigorous statistical assessment. During group discussions and interviews, for both HWS group categories, study participants explained that the main motivation to wash hands with soap was child’s health and having water and soap in one place near the food preparation and child feeding area facilitated the practice. Mothers explained that nutritious food that they were providing to their children will not provide nourishment if they do not wash their hands before feeding their child. For example one mother from the self-made HWS group said- "*If I do not wash my hands with soap then all germs remain on my hands and my child’s food will be contaminated. Therefore these germs will enter my child’s stomach and it may cause my child’s illness. For my child’s wellbeing I wash my hands with soap".* Regarding increasing handwashing behavior one mother from study-HWS group said- “*Before getting this handwashing station I needed to go to the tube well for washing hands; therefore I didn’t wash my hands sometime because I was not motivated. Now I get water and soap together close to my hands; so I always wash my hands with soap.”* For both intervention groups mothers reported that they forgot to wash their hands due to the pressure of household chores. In the self-made HWS group another barrier was that sometimes water and soap were not easily accessible. For example one caregiver explained that *"My husband is a farmer and sometimes he takes the water vessel to the farmland for cultivation purposes, and we don't have another suitable vessel that I can use to keep at the designated place. In that case sometimes I forgot to wash hands before food preparation"*.

### Regular use and maintenance of handwashing stations

At the final assessment 90% (9/10) of households in the study-HWS group and 60% (6/10) households in the self-made HWS group were observed to have a complete handwashing station (both water and soap/soapy-water) that was functional and caregivers used at least once during the observation period. However, soapy-water was detected among 100% of the observed households in both HWS groups.

### Motivators and barriers to regular use and maintenance of handwashing stations

Respondents from both study groups reported that a handwashing station was easy to use, and that the presence and placement of the handwashing station motivated people to regularly use and maintain it, encouraging handwashing at promoted times. Study participants explained that having water and soap in one place near food preparation and child feeding areas reduced the need to go to a distant, inconvenient location to wash hands with soap. For example one caregiver explained that “*handwashing station makes my handwashing easier because we get water and soap near my cooking place and no need to go to the tube well frequently. I can wash my hands by sitting at my cooking place.”* Another caregiver said “*We have no tubewell at our home. Before getting this handwashing station we had to go outside (to a common tube well for several households) for washing our hands and utensils. Moreover many households use one tube well and no one keeps soap beside tube well. Now we get water and soapy-water together and we don’t need to go outside and we can wash our hands whenever we need".*

Similarly, caregivers in the self-made HWS group described the advantages and benefits of keeping water and soap together and close at hand as they didn’t need to go far if they maintain a stocked handwashing station near the food preparation and child feeding area(s). Community members of both intervention groups reported a preference for soapy-water over bar soap as it is easier to use than bar soap.

During the interviews caregivers in the study HWS group also reported that they liked the appearance and free provision of the handwashing station and that it acted as a reminder to wash hands.

Study participants reported some strengths of the handwashing stations that influenced regular use. Study participants in the study HWS group explained that the tap on the bucket increased convenience in providing running water while washing their hands. They reported that the station facilitated handwashing among children providing readily available water by turning the tap, compared to the alternative where they need to pump water from tube wells, which can be strenuous and thus act as a barrier to handwashing. Although, in the self-made HWS group nobody had a tap on the water vessel they arranged, they used a mug to pour water over hands. Study participants reported that this arrangement had an advantage of the tap. They explained that, "*we could not set up a tap with our water vessel by ourselves because we need to spend money to buy a tap to hire a mechanic to set it on the bucket. Also the tap can become cracked after a certain moment*".

The bowl with the study-provided bucket was considered a strength as it held residual rinse water and reduced the risk of water remaining on the floor. Many participants in the self-made HWS group placed a bowl at their own handwashing station for this purpose. In addition, the lid on the study provided bucket kept water clean and the study participants reported that children can’t waste water when the lid is kept locked. They also reported that the lid enhanced safety by preventing children from falling into the water. On the other hand, lack of a lid for the self-made handwashing stations acted was considered a weakness. Study participants from the self-made HWS group mentioned that their handwashing water vessel that was uncovered encouraged children to play with the water. None from the self-made HWS group used an existing bucket that had a lid. Caregivers feared that playing with water may cause a child to develop a cough or a cold. They also described a risk for young child to fall into the uncovered water vessel. Although, the main reported barrier for both intervention group categories was wastage as children frequently play with the water and soap/soapy-water which was more frequently reported by respondents from the self-made HWS group.

Participants from the study HWS group liked the large size of the bucket they received, so that they could store water for all household members for a full day. They reported that they kept the handwashing station inside their living room at night so that they can wash their hands whenever necessary. For example one mother said, "*My son and his father can also wash their hands from this handwashing station. Now we don’t need to go outside at night for washing hands and utensils.”* However, in the self-made HWS group field researchers didn’t observe any water vessel of a similar size to the bucket provided with model handwashing station. Study participants from both groups explained that the handwashing station was convenient for other purposes related to cooking and feeding/eating, not just for handwashing.

Another strength of handwashing intervention was provision of soapy-water. Dispensing soapy-water from the bottle with a hole in the cap onto the palm of their hands reduced the duration of handwashing and this product was less likely to be wasted by children. For example one mother from Manikgonj said, *“Our tube well is located so far and in an open place. If I keep soap (bar soap) in that place, sometimes a crow will take away the soap, and sometimes it becomes spoiled. But the soapy-water is better than soap. It’s easy to use, and is less likely to be damaged.”* Caregivers in the self-made HWS group reported that soapy-water was inexpensive to make; most of the observed households from this group made their own soapy-water, even though they didn’t receive a bottle or detergent. For example, one caregiver from Dinajpur said, *"Soap (bar soap) is expensive. Detergent powder remains available in my home all the time for washing my clothes; and I can use a small amount for making soapy-water. So I don't need to expend extra money for making soapy-water. Even some of my neighbors prepared and are using soapy-water by their own initiative even though they don't have children under 2 years of age (were not part of the intervention)."* This was detected during the final assessment, when field researchers observed neighbors that were not included in promotion activities prepared their own soapy-water.

### Understanding IYCF messages:

The majority of participants (caregivers) recalled intervention messages on social and health benefits of infant and young child feeding correctly. They described appropriate frequency, amount and variety of complementary food and how this helps a child’s physical and mental growth. They also reported the health risk if they did not follow recommended practices. In addition to the IYCF messages, all of the caregivers recalled the importance of handwashing with soap before food preparation and feeding a child. One caregiver explained that *"we are giving good food to my child for his good health, but if we do not wash our hands before feeding him then the food will not work at all"*.

## Discussion

In Bangladesh, preparing foods and feeding a child with bare hands is common. Food preparation and feeding a child with unwashed hands can contaminate food and can be a source of diarrheal pathogens [25, 26]. A study conducted in Bangladesh reported that 40% of complementary foods were contaminated with pathogenic bacteria [13]. Washing hands with soap before food preparation can significantly reduce the incidence of childhood diarrhea [14]. However, in Bangladesh caregivers don’t commonly wash their hands with soap before food preparation or before feeding a child [18] thereby increasing the risk of food contamination. Verplanken and Wood [27] suggest that for a habit formation, repeated behavior in a stable context is required. A handwashing station may act as the stable context for habit formation and may facilitate the handwashing behavior by placing water and soap together at the convenient locations [28, 29]. The current study findings indicate that households can develop and use their own devices to keep water and soap available at the handwashing place if encouraged to do so. Among a small number of observations it seems that encouraging device placement through repeated home visits resulted in handwashing with soap at the recommended times over the short term. This is consistent with a study conducted in Bangladesh that concluded that handwashing with soap improved when water and soap were present at locations where handwashing took place [17].

Among households from the self-made HWS group, more than half of them were observed to have a handwashing station at a place convenient to cooking and child feeding even when no products were provided. However, those who received study provided handwashing stations were more likely to have them placed convenient to cooking and child feeding. We observed that many mothers who were not supplied with hardware were sufficiently motivated to develop and place their own handwashing station and prepare soapy water. It is likely that having a water source close to the house, near the cooking/feeding area(s) increased convenience, not only for handwashing but for other domestic tasks. Soapy-water was a popular handwashing agent as it was viewed as being less expense than bar soap, easy to maintain and less likely to be wasted, as described among hardware recipients previously [24].

Among the self-made HWS group, many of the caregivers used hardware that was not dedicated for handwashing and could be removed for other purposes thereby leaving the cooking area without a water source. Engaging male members of the family while explaining the importance of maintaining the handwashing stations in the specified location is an important step. Future interventions that promote development of handwashing stations from households’ resources could address this by providing guidance on hardware composition and stressing the convenience of dedicating the hardware for handwashing and food preparation. The handwashing stations developed by household members did not have a lid and participants described their concerns of having open buckets of water in the household area and the danger that presented. A lid can address concerns about the potential for water wastage, concerns about children catching a cold and prevent children falling into the water. Future program should include not only dedicated devices for self-made handwashing station, but promoting the advantages of a lid and encourage purchase and use of a cover to reduce caregiver concerns. The tap, while useful, was not seen as essential, therefore a bucket with lid and jug would be a low cost, readily available model. Also, lack of facilities to capture waste water made the courtyard wet and muddy, a potential reason for lower observed handwashing with soap in the self-made HWS group. Hence, recommendation for a container to collect waste water should be included as a component of self-made handwashing station. However, an encouragingly large number of households were observed to place self-made handwashing station and soapy water bottles through behavior change promotion. Preparing and placing a soapy-water bottle by those outside the promotion audience indicates that this is likely a feasible solution for keeping soap near water (for other key handwashing times such as post defecation), and near cooking and feeding areas.

Stunting is a marker of chronic malnutrition and is associated with an increased risk of diarrhea. Integration of handwashing into a nutrition program has the potential to prevent diarrheal diseases. Our study suggests that it is possible to integrate handwashing messages into an IYCF intervention. The majority of the study participants understood the importance of complementary food and could connect the link between unwashed hands and food contamination which ultimately increased their handwashing behavior. We suggest further research to investigate the potential impact of an integrated handwashing intervention and child feeding intervention on the occurrence of diarrhea.

Our study also suggests that CHWs have a positive impact on improving handwashing behavior. This finding is consistent with other studies that concluded that CHWs can improve health-service use and child health outcomes [30]. However, the current study was too short term to assess the duration of CHW exposure required among the target communities to achieve sustained habit adoption.

A limitation of the study is that we conducted the study in only two rural sites, however, these were typical of rural Bangladeshi communities. As it was a pilot study it was conducted on a small scale for a short period, yet it provided useful insights for future interventions. Sustained practice can be assessed during longer duration trials.

## Conclusion

Encouraging caregivers to create and use their own handwashing station was feasible and effective in increasing handwashing behavior before food preparation and before feeding a child. In the absence of large-scale provision of handwashing stations, caregivers can be encouraged to create and use their own. Soapy water was a popular alternative to bar soap and should be considered for promotion at scale. Strategies to promote own handwashing station placement should includes motivation through behavior change communication, creating a designated place for handwashing at the recommended key times, through regular home visits by community health workers that provide supports in designing own handwashing stations by using available household resources. Integrating handwashing with soap into a nutrition intervention was feasible and acceptable and should be considered by policy makers.
